# Supplementary material for: Early Cambrian fuxianhuiids from China reveal origin of the gnathobasic protopodite in euarthropods
Source: Nat Commun. 2018 Feb 1;9:470. doi: 10.1038/s41467-017-02754-z (PMC5794847; doi:10.1038/s41467-017-02754-z)
Supplement: Supplementary file 1 — Supplementary Information [file 41467_2017_2754_MOESM1_ESM.pdf]

Supplementary Information

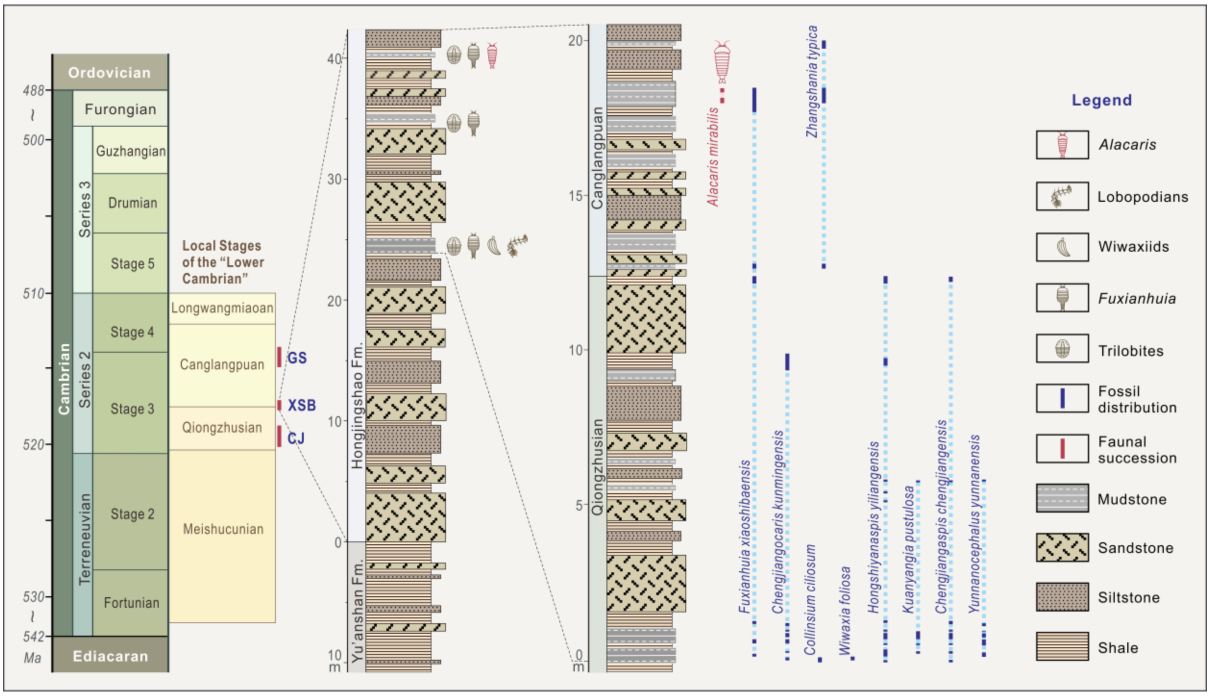

Supplementary Figure 1 | Stratigraphic column and fossil distribution within the Cambrian (Stage 3) Hongjingshao Formation, Xiaoshiba section, Kunming. *Alacaris mirabilis* is found in mudstone about 10 meters above the fossil assemblage of *Yunnanocephalus–Chengjiangaspis–Hongshiyanaspis* biozone[1–3]. According to the coexisting trilobite *Zhangshania*, *Alacaris mirabilis* occurs at the bottom of the Canglangpuan. Geologically the horizon of the Xiaoshiba Lagersttäte (XSB) is above the Chengjiang (CJ) and below the Guanshan (GS) biotas.

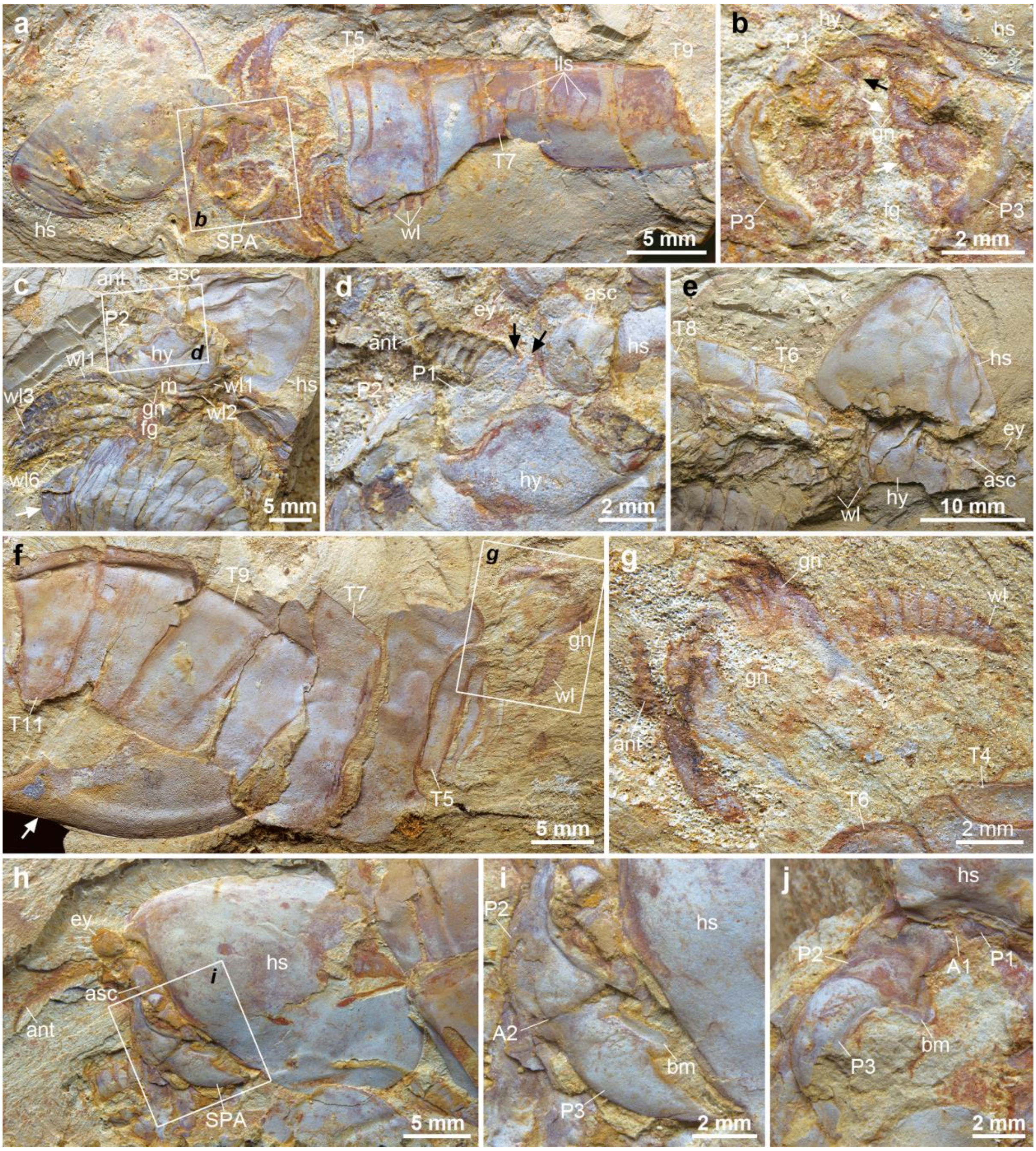

Supplementary Figure 2 | *Alacaris mirabilis* from the Cambrian (Stage 3) Xiaoshiba Lagerstätte. (**a**) YKLP 12276, ventral view of a partially disarticulated specimen showing the anterior appendicular organization. (**b**) Close-up of area *b*, showing overall organization of the SPAs, fine spinose endites along the inner basal margin of the SPAs (black arrow) and gnathobases of the post-oral appendages (white arrow). (**c**) YKLP 12310a, anterior portion of individual preserved in ventral view ventral view, buried alongside articulated tergites of *Fuxianhuia* sp. (white arrow). (**d**) Close-up of area *d*, showing stalked eyes, antenna and hypostome. (**e**) YKLP 12310b, counterpart showing fragmentary head shield and tergites. (**f**) YKLP 12311, anterior portion of an incomplete individual together, preserved alongside a trilobite librigena (arrowed). (**g**) Close-up of area *g*, showing antenna and gnathobases with strong spinose endites. (**h**) YKLP 12272, anterior portion of an individual preserved in lateral view showing stalked eye, an antenna, and the left SPA. (**i**) Enlargement of area *i*, showing details of the SPA, which is ornamented with fine nods, showing articulation and marginal

blade. (**j**) YKLP 12273, a SPA showing blade-like inner margin and articulation. Abbreviations: A*n*, articulations; ant, antenna; asc, anterior sclerite; bm, blade-like margin; ey, eye; fg, food groove; gn, gnathobase; hs head shield; hy, hypostome; ils, impression of leg sheath; m, mouth; P*n*, podomeres; SPA, specialized post-antennal appendage; T*n*, tegites; wl*n*, walking legs.

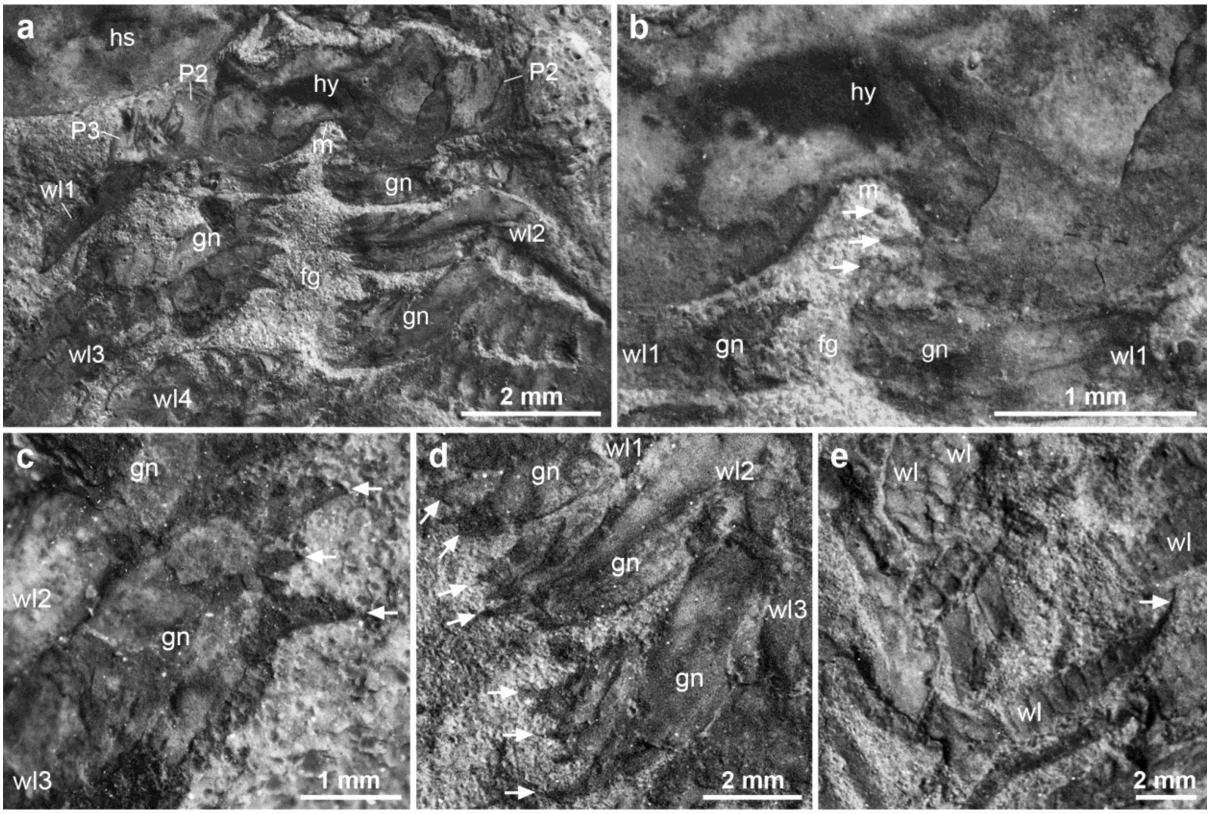

Supplementary Figure 3 | Composite-fluorescence illustrations of feeding apparatus in *Alacarus mirabilis* from the Cambrian (Stage 3) Xiaoshiba Lagerstätte. (**a**) YKLP 12268 (holotype), ventral view revealing the hypostome overlying the basal portions of the SPAs, and the differentiated gnathobasic protopodite with strong spinose endites. (**b**) Close-up of (a), showing posterior notch in the hypostome that most likely accommodated the posterior-facing mouth opening; note the preservation of spinose endites (white arrow) of the SPAs underneath the hypostome. (**c**) Details of (**a**) showing the fine spinose endites of varying in size (arrowed) of the third head limb on the right side. (**d**) Well-preserved gnathobases on the left side. (**e**) YKLP 12275, detail of walking legs with a long distal spine (arrowed). Abbreviations: as in Supplementary Figure 2.

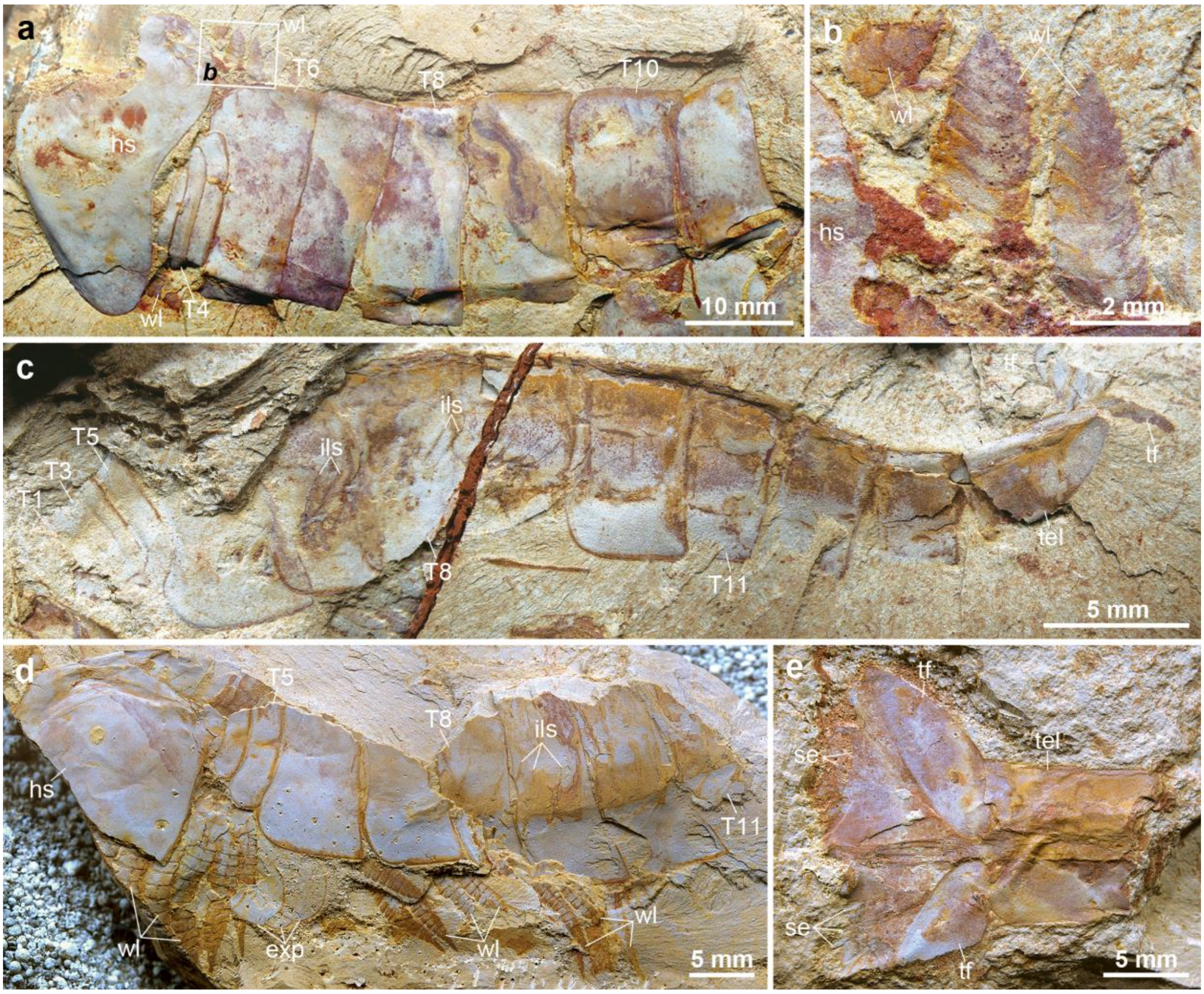

Supplementary Figure 4 | Dorsal exoskeleton of *Alacaris mirabilis* from the Cambrian (Stage 3) Xiaoshiba Lagerstätte. (**a**) YKLP 12271, large individual with slightly displaced cephalic shield and articulated trunk tergites. (**b**) Close-up of area *b*, showing anterior walking legs on the right side. (**c**) YKLP 12277, complete specimen preserved in lateral view, showing full tergite count; note the presence of five anterior reduced tergites. The leg sheath impressions indicate the presence of several pairs of limbs per tergite, and suggest the absence of limbs on the posterior tergites. (**d**) YKLP 12312, anterior portion of a fragmentary individual showing preserved endopods and exopods of walking limbs. (**e**) YKLP 12268b, counterpart of the type specimen with only the paired tail flukes preserved. Abbreviations: se, setae; tel, tail spine; tf, tail fluke; others as in Supplementary Figure 2.

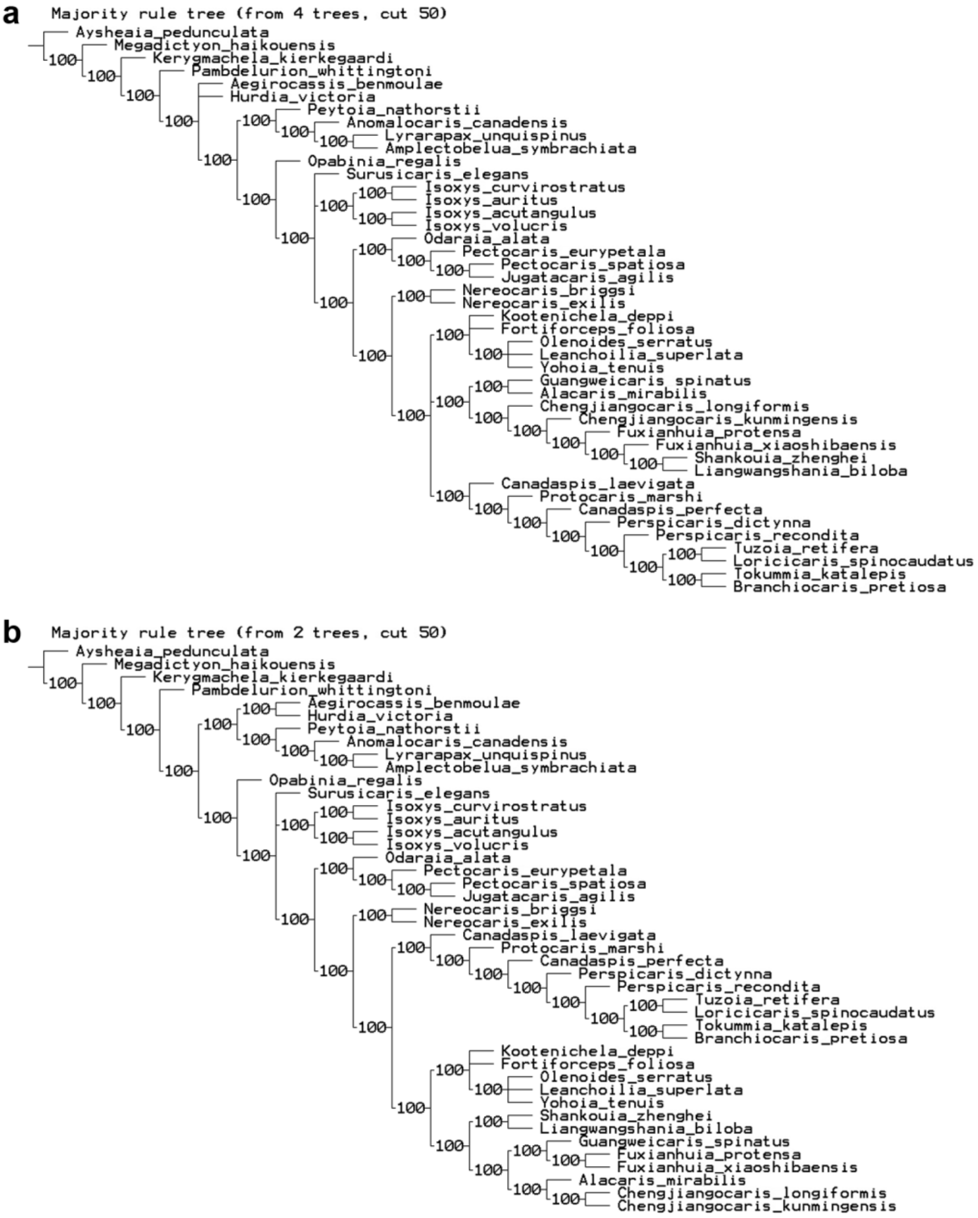

Supplementary Figure 5 | Phylogenetic position of *Alacaris mirabilis* in the context of total-group Euarthropoda. (**a**) Majority rule consensus of four most parsimonious trees calculated under equal weights. (**b**) Majority rule consensus of two most parsimonious trees calculated under implied weights (k=3).

Supplementary Note 1 | Systematic Palaeontology

(upper stem-group) Euarthropoda Lankester, 1904 (see discussion in ref.4)

Fuxianhuiida Bousfield,1995[5]

Constituent taxa. *Shankouia zhenghei* Walozsek *et al.*, 2005[6] (Cambrian Stage 3, Chengjiang); *Liangwangshania biloba* Chen, 2005[7] (Cambrian Stage 3, Chengjiang); Fuxianhuiidae Hou and Bergström, 1997[8] (Cambrian Stage 3, Chengjiang, Xiaoshiba and Guanshan); Chengjiangocarididae Hou and Bergström, 1997[8] (Cambrian Stage 3, Chengjiang and Xiaoshiba).

Emended diagnosis. Euarthropods with unfused subtrapezoidal head shield articulated with an anterior sclerite associated with stalked compound eyes. Trunk consists of broadly overlapping tergites that taper in width posteriorly. Variable number of anteriormost reduced tergites concealed under head shield in life position. Pre-oral first appendage pair antenniform, composed of up to 20 podomeres. Para-oral second appendage pair robust, consisting of three podomeres with acute distal termination. Sclerotized hypostome covers mouth opening as proximal bases of second limb pair. Trunk limbs biramous, with homonomous construction throughout body. Up to four limb pairs are associated with each trunk tergite. Endopod consists of approximately 12 podomeres. Differentiated gnathobasic protopodite confirmed only in some species. Exopod oval-shaped, fringed with short marginal setae. Tail spine conical or paddle-shaped, frequently associated with paired tail flukes. Revised from ref. 8.

Remarks. The monophyly of Fuxianhuiida Bousfield, 1995[5] is supported by several synapormorphies, and further confirmed by the results of the phylogenetic analysis (Supplementary Fig. 5). The proposed emended diagnosis provides a more accurate depiction of the morphological characters that define Fuxianhuiida relative to previous studies that have addressed the classification of this clade[5,8]. *Shankouia zhenghei* Waloszek*et al.*, 2005[6], and *Liangwangshania biloba* Chen, 2005[7], cannot be accommodated into either of the existing families based on their morphology (*contra* ref. 7). Although both of these taxa possess an elongate trunk with expanded tergopleurae that taper posteriorly and a paddle-shaped tail spine, the results of the phylogenetic analysis does not support their monophyly into a clade (Supplementary Fig. 5), but rather as basally branching members within Fuxianhuiida.

Fuxianhuiidae Hou and Bergström, 1997[8]

= Guangweicarididaec Luo *et al.*, 2007[9]

Constituent taxa. *Fuxianhuia protensa*Hou, 1987[10] (Cambrian Stage 3, Chengjiang); *Fuxianhuia xiaoshibaensis* Yang *et al.*, 2013[2] (Cambrian Stage 3, Xiaoshiba); *Guangweicaris spinatus* Luo *et al.*, 2007[9] (Cambrian Stage 3, Guanshan).

Emended diagnosis. Fuxianhuiids with subtrapezoidal head shield with approximately 1:4 length/width ratio, covering three anteriormost reduced tergites. Trunk subidivided into two morphologically distinct regions, consisting of anterior limb-bearing thorax with well-developed expanded tergopleurae, and narrow limb-less abdomen with ring-like tergites. Endopods of biramous limbs with rounded termination; endopods short in length, not reaching beyond the thoracic tergite and head shield margins.

Remarks. The close morphological similarities shared between *Fuxianhuia* species[2,8,11] and *Guangweicaris*—particularly the presence of three reduced tergites and the differentiation of the trunk into a thoracic and abdominal regions—support their classification within Fuxianhuiidae. We follow Yang[12] in recognizing Guangweicarididae Luo *et al.*, 2007[9], as a synonym of FuxianhuiidaeHou and Bergström, 1997[8].

Chengjiangocarididae Hou and Bergström, 1997[8]

Constituent taxa. *Chengjiangocaris longiformis* Hou and Bergström, 1991[13] (Cambrian Stage 3, Chengjiang); *Chengjiangocaris kunmingensis* Yang *et al.*, 2013[2] (Cambrian Stage 3, Xiaoshiba); *Alacaris mirabilis* gen. et sp. nov. (Cambrian Stage 3, Xiaoshiba).

Emended diagnosis. Fuxianhuiids with subtrapezoidal heart-shaped head shield with approximately 1:1 length/width ratio, covering five anteriormost reduced tergites. Trunk tergites gradually taper in width posteriorly. Biramous limbs present throughout most of trunk, except for posteriormost tergites. Endopod of biramous limbs well-developed, terminating in conical tip. Enodpods elongate, frequently reaching beyond trunk tergite and head shield margins.

Remarks. *Alacaris mirabilis*is confidently recognized as a member of Chengjiangocarididae Hou and Bergström[8] based on the presence of five anteriormost reduced trunk tergites, the dimensions of the head shield, a posterior tapering trunk, and the morphology of the endopods. The main differences between *Alacaris* and *Chengjiangocaris* species[2,8,13] are expressed in the number of trunk tergites and the presence of a well-developed protopodite in the former taxon.

Supplementary Note 2 | Character coding

Continuous characters

Cephalic (carapace)

0. *Length to width ratio of anterior sclerite.*

Inapplicable for taxa lacking a distinct anterior sclerite (Character 17). An elongate anterior sclerite, compared to width, is seen in the hurdids, specifically *Hurdia victoria*, and *Aegirocassis benmoulae*, whilst the anomalocaridids, and upper-stem-group euarthropods have an anterior sclerite that is wider than long.

1. *Tallest point of carapace.*

Inapplicable for taxa lacking a carapace (Character 24). There is a lot of variety of this character amongst “carapace-bearing arthropods”, with isoxyids typically possessing a shallow carapace posterior with the tallest point occurring in the anterior half of the domicilium, whist protocaridids are more rounded, with the tallest point occurring towards the middle, and fuxianhuids towards the posterior of the carapace.

2. *Height to length ratio of carapace domiciluium.*

Inapplicable for taxa lacking a carapace (Character 24). This character serves to distinguish the carapaces of most “carapace-bearing arthropods”, in which the carapace is typically longer than wide, from fuxianhuids, in which the carapace is wider than long.

3. *Length of antero-dorsal carapace spines.*

Inapplicable for taxa lacking antero-dorsal carapace spines (Character 29). This character specifically refers to species of *Isoxys*. Both *I. volucris*, and *I. curvirostratus* possess an elongate antero-dorsal spine, whilst that of *I. acutangulus* and *I. auritus* is much shorter.

4. *Length of postero-dorsal carapace spines.*

Inapplicable for taxa lacking postero-dorsal carapace spines (Character 29). This character specifically refers to species of *Isoxys*.

5. *Slope of posterior carapace margin.*

Inapplicable for taxa lacking a carapace (Character 24). This character serves to distinguish *Isoxys* from other species of “carapace-bearing arthropods”. *Isoxys* is unique in possessing a deeply sloped posterior carapace margin.

Meristic characters

Meristic characters were analysed as continuous characters. This differs from previous analyses that have treated them as discrete, thereby creating arbitrary bins and reducing character linkage and hindering trait determination.

Trunk

6. *Number of trunk segments*.

This character could not be determined for many taxa bearing a carapace, as this feature frequently covers an anterior series of poorly sclerotized trunk segments (e.g. *Nereocaris, Loricicaris*). Likewise, this character was not coded for the trilobite *Olenoides serratus*, as the exact number of segments in the pygidium could not be determined.

7. *Number of prothoracic segments*.

This character is inapplicable for taxa lacking a prothorax (Character 42). The fuxianhuidids, namely *Guangweicaris spinatus*, *Fuxianhuia protensa*, and *F. xiaoshibaensis*, possess three prothoracic segments, whilst the chengjiangocaridids, *Alacaris mirabilis*, *Chengjiangocaris longiformis*, and *C. kunmingensis*, possess five, and *Shankoia zhenghei* possesses six. This character could not be adequately determined for *Liangwangshania biloba* as there is little variation between the morphology of the posterior prothoracic segments and the anterior post-prothoracic segments.

8. *Number of post-prothoracic thorax segments*.

This character is inapplicable for taxa lacking a prothorax (Character 42). Unfortunately, the extent of the thorax could not be determined in a number of fuxinhuids as the position of the appendage, which would serve to delineate the thorax in taxa without a posteriorly differentiated abdomen, could also not be determined. *Guangweicaris spinatus* possesses a short, five segmented, post-prothoracic thorax, whilst the two species of *Fuxianhuia* both possess 17.

9. *Number of abdominal segments*.

This character is inapplicable for taxa lacking a distinct abdomen delineated by either a lack of appendages, or a notable change in dimension from the anterior (thoracic) trunk segments.

Appendicular (cephalic)

10. *Number of podomeres in pre-antennular (protocerebral) appendages*.

This character is inapplicable for taxa lacking an arthropodized protocerebral appendage with distinct podomeres (Character 50).

11. *Number of post-protocerebral cephalic appendage pairs in cephalic region*.

This character is inapplicable for taxa lacking a distinct sclerotized cephalic covering, and could not be determined in most “carapace-bearing arthropods” due to uncertainty regarding the position of the cephalon-trunk boundary. In fuxianhuids this can be determined based on the position of the prothorax relative to the anterior appendages.

12. *Number of podomeres in differentiated tritocerebral appendage*.

This character is inapplicable for taxa lacking a pair of differentiated tritocerebral appendage (Character 61). All fuxianhuids for which a differentiated tritocerebral appendage is known possess three segments in each appendage, whilst megacheirans possess either five or six, and the protocaridids, *Branchiocaris pretiosa* and *Tokumnia katalepis* both possess eight.

13. *Number of spine-bearing distal podomeres on tritocerebral appendage.*

This character is inapplicable for taxa lacking spinose projections on a differentiated tritocerebral appendage (Character 61).

Appendicular (general)

14. *Number of exite segments*.

This character is inapplicable for taxa lacking exites (Character 65). This character serves to distinguish advanced megacheirans, specifically *Yohoia tenuis* and *Leanchoilia superlata*, and the trilobite *Olenoides serratus*, which possess an exite composed of two segments, from the other megacheirans, and stem-lineage euarthropods, including the dinocaridids, and “carapace-bearing arthropods”, which possess just a singular segment.

15. *Number of trunk endopod segments (podomeres).*

Appendicular (lateral processes)

16. *Number of lateral process pairs*.

Discrete characters

Cephalic (including external ocular features)

17. *Anterior (ocular) sclerite: (0) absent, (1) present*.

18. *Anterior margin of anterior sclerite tapered: (0) absent, (1) present*.

19. *Anterior sclerite flanked by lateral plates: (0) absent, (1) present*.

20. *Lateral eyes: (0) absent, (1) present*.

21. *Lateral eyes stalked: (0) absent, (1) present*.

22. *Single medial eye: (0) absent, (1) present*.

23. *Sclerotized cephalic covering: (0) absent, (1) present*.

Cephalic (carapace)

24. *Free posterolateral extensions of sclerotized cephalic covering: (0) absent, (t) present*.

25. *Antero-ventral and postero-dorsal margins of carapace parallel: (0) absent, (1) present*.

26. *Carapace reticulate: (0) absent, (1) present*.

27. *Reticulation type: (0) small mesh, (1) large, hexagonal, mesh*.

28. *Anterior extension of carapace: (0) absent, (1) present*.

29. *Anterodorsal spine: (0) absent, (1) present*.

30. *Anteroventral hook-like processes: (0) absent, (1) present*.

31. *Posterodorsal keel: (0) absent, (1) present*.

32. *Carapace divided medial by suture or hinge: (0) absent, (1) present*.

33. *Posteromedial suture on carapace: (0) absent, (1) present*.

34. *Posterodorsal spine: (0) absent, (1) present*.

35. *Lateral margins of carapace contiguous with lateral pleural trunk margins: (0) absent, (1) present.*

Trunk

36. *Sclerotized trunk: (0) absent, (1) present*.

37. *Paired dorsal nodes: (0) absent, (1) present*.

38. *Dorsal band of blade-like setae: (0) absent, (1) present*.

39. *Pleural extension oftergites: (0) absent, (1) present*.

40. *Posterolateral margins of pleurae extended into spinose projections: (0) absent, (1) present.*

41. *Raised axial region: (0) absent, (1) present*.

42. *Anterior trunk segments greatly reduced forming a prothorax: (0) absent, (1) present*.

43. *Abdomen differentiated as limb-free segments: (0) absent, (1) present*.

44. *Abdomen differentiated as posteriorly-restricted segments: (0) absent, (1) present*.

45. *Posterior trunk segments with spinose posterior rim: (0) absent, (1) present*.

46. *Penultimate trunk segment elongate: (0) absent, (1) present*.

Telson

47. *Telson: (0) absent, (1) present.*

Appendicular (pre-antennular, protocerebral)

48. *Protocerebral appendages: (0) absent, (1) present.*
49. *Orientation of protocerebral appendages: (0) lateral, (1) ventral.*
50. *Sclerotization of protocerebral appendages: (0) absent, (1) present.*
51. *Fusion of protocerebral appendages: (0) absent, (1) present.*
52. *Hypostome with lateral slits: (0) absent, (1) present.*
53. *Protocerebral appendages spinose: (0) absent, (1) present.*
54. *Dorsal spine row: (0) absent, (1) present.*
55. *Ventral spine row: (0) absent, (1) present.*
56. *Relative orientation of dorsal spine row to podomere: (0) perpendicular, (1) lateral (chelate).*
57. *Elongate ventral basal spine: (0) absent, (1) present.*
58. *Secondary (auxillary) spines on ventral spine row: (0) absent, (1) present.*
59. *Flexure of terminal protocerebralpodomere: (0) ventral, (1) dorsal.*

Appendicular (antennular, deutocerebral)

60. *Deutocerebral appendage differentiated from posterior appendages: (0) absent, (1) present.*

Appendicular (First-post-antennular, tritocerebral)

61. *Tritocerebral appendage differentiated from posterior appendages: (0) absent, (1) present.*
62. *Basal podomere bearing endites: (0) absent, (1) present.*
63. *Appendages geniculate with distinct peduncle: (0) absent, (1) present.*
64. *Chelate (or subchelae) distal podomeres: (0) absent, (1) present.*

Appendicular (post-tritocerebral)

65. *Exites: (0) absent, (1) present.*
66. *Longitudinal wrinkling on exites: (0) absent, (1) present.*
67. *Exite fringed with setae: (0) absent, (1) present.*

68. *Exite and endopod derived from the same parent podomere (biramy): (0) absent, (1) present*.

69. *Trunk endopods: (0) absent, (1) present*.

70. *Endopod shape: (0) leg-like, (1) flap-like*.

71. *Sclerotization and arthropodization of trunk endopods: (0) absent, (1) present*.

72. *Endites on endopod: (0) absent, (1) present*.

73. *Endites on basal endopod segment: (0) absent, (1) present*.

74. *Endopod with terminal claws: (0) absent, (1) present*.

Appendicular (lateral processes)

75. *Posterior tagmata with elongate lateral processes: (0) absent, (1) present*.

76. *Lateral process pairs fused into a single element retaining evidence of original segmentation: (0) absent, (1) present*.

77. *Shape of lateral processes: (0) bulbous (unsclerotized) flaps, (1) cerci, (2) sub-triangular, (3) paddle-like*.

78. *Lateral telson processes recurved: (0) absent, (1) present*.

79. *Lateral telson processes spinose: (0) absent, (1) present*.

Digestive

80. *Orientation of mouth: (0) anterior, (1) ventral, (2) posterior*. [ADDITIVE]

81. *Circumoral structures: (0) absent, (1) present*.

82. *Nature of circumoral structures: (0) papillae, (1) sclerotized plates*.

83. *Lateral gut glands: (0) absent, (1) present*.

Supplementary References

1. Yang, J., *et al.* A superarmored lobopodian from the Cambrian of China and early disparity in the evolution of Onychophora. *Proc. Natl. Acad. Sci., U.S.A.* **112**, 8678–8683 (2015).

2. Yang, J., Ortega-Hernández, J., Butterfield, N. J. & Zhang, X.-G. Specialized appendages in fuxianhuiids and the head organization of early euarthropods. *Nature* **494**, 468–471 (2013).

3. Li, S.-J., Kang, C.-L. & Zhang, X.-G. Sedimentary environment and trilobites of Lower Cambrian Yuxiansi Formation in Leshan District. *Bull. Chengdu Inst. Geol. Min. Resour., Chinese Acad. Geol. Sci.* **12**, 37–56 (1990) [in Chinese].

4. Ortega-Hernández, J. Making sense of 'lower' and 'upper' stem-group Euarthropoda, with comments on the strict use of the name Arthropoda von Siebold, 1848. *Biol. Rev.* **91**, 255–273 (2016).5. Bousfield, E. I. A contribution to the natural classification of Lower and Middle Cambrian arthropods: food gathering and feeding mechanisms. *Amphipacifica* **2**, 3–34 (1995).

6. Waloszek, D., Chen, J.-Y., Maas, A. & Wang, X.-Q. Early Cambrian arthropods—new insights into arthropod head and structural evolution. *Arthropod Struct. Dev.* **34**, 189–205 (2005).

7. Chen, A.-L. A new *Fuxianhuia*-like arthropod of the early Cambrian Chengjiang fauna in Yunnan. *Yunnan Geol.* **24**, 108–113 (2005).

8. Hou, X.-G. & Bergström, J. Arthropods of the Lower Cambrian Chengjiang fauna, southwest China. *Fossils Strata* **45**, 1–116 (1997).

9. Luo, H.-L., Fu, X., Hu, S.-X., You, T., Pang, J. & Liu, Q. A new arthropod *Guangweicaris* gen. nov. (Luo, Fu *et* Hu) from the Early Cambrian Guanshan fauna, Kunming, China. *Acta Geol. Sin.* **81**, 1–7 (2007).

10. Hou, X.-G. Three new large arthropods from Lower Cambrian, Chengjiang, eastern Yunnan. *Acta. Palaeontol. Sin.* **26**, 272–285 (1987).

11. Bergström, J., Hou, X.-G., Zhang, X.-G., Liu, Y. & Clausen, S. A new view of the Cambrian arthropod *Fuxianhuia*. *GFF* **130**, 189–201 (2008).

12. Yang, J., Hou, X.-G. & Dong, W. Restudy of *Guangweicaris* Luo, Fu et Hu, 2007, from the Lower Cambrian Canglangpu Formation in Kunming area. *Acta Palaeontol. Sin.* **47**, 115–122 (2008).

13. Hou, X.-G. & Bergström, J. The arthropods of the Lower Cambrian Chengjiang fauna, with relationships and evolutionary significance. in *The Early Evolution of Metzoan and the Significance of Problematic Taxa* (eds Simonetta, A. M. & Conway Morris, S.) 179–187 (Cambridge Univ. Press, Cambridge 1991).
